# Nosocomial Outbreak of Crimean-Congo Hemorrhagic Fever, Sudan

**DOI:** 10.3201/eid1605.091815

**Published:** 2010-05

**Authors:** Imadeldin E. Aradaib, Bobbie R. Erickson, Mubarak E. Mustafa, Marina L. Khristova, Nageeb S. Saeed, Rehab M. Elageb, Stuart T. Nichol

**Affiliations:** The National Ribat University, Khartoum, Sudan (I.E. Aradaib); University of Khartoum, Khartoum (I.E. Aradaib); Centers for Disease Control and Prevention, Atlanta, Georgia, USA (B.R. Erickson, M.L. Khristova, S.T. Nichol); Central Public Health Laboratory, Khartoum (M.E. Mustafa); Juba University, Khartoum (M.E. Mustafa); Federal Ministry of Health, Khartoum (N.S. Saeed); National Medical Laboratory, Khartoum (N.S. Saeed, R.M. Elageb)

**Keywords:** Nosocomial outbreaks, viral hemorrhagic fevers, CCHF virus, S segment, Sudan, viruses, dispatch

## Abstract

To confirm the presence of Crimean-Congo hemorrhagic fever in Sudan, we tested serum of 8 patients with hemorrhagic fever in a rural hospital in 2008. Reverse transcription–PCR identified Crimean-Congo hemorrhagic fever virus. Its identification as group III lineage indicated links to virus strains from South Africa, Mauritania, and Nigeria.

Crimean-Congo hemorrhagic fever virus (CCHFV; family *Bunyaviridae*, genus *Nairovirus*) is a tick-borne virus. Its tripartite RNA genome consists of small (S), medium, and large segments. The virus is distributed throughout much of Africa, Asia, and southern Europe (*1*–*5*). In some regions, the virus is responsible for annual outbreaks of hemorrhagic fever with high case-fatality rates; in others, it causes sporadic cases only. Because of its association with rapid-onset hemorrhagic fever and an ≈30% case-fatality rate, CCHFV is on the US Select Agent list of agents considered to have bioterrorism potential (*2*–*5*).

Distribution of CCHF largely mirrors that of its Ixodid tick hosts, particularly those of the genus *Hyalomma* (*1*). Persons become infected when bitten by virus-infected ticks or after contact with blood or tissue from viremic livestock or other persons. Outbreaks often involve persons in rural communities, such as shepherds, slaughterhouse workers, or medical staff of resource-poor hospitals. Despite presence of *Hyalomma* tick vectors in Sudan, no CCHF cases have been confirmed there. However, in the past 2 years, suspected CCHF outbreaks and sporadic cases in the Kordufan region of Sudan have been reported.

From a public health perspective, confirming CCHF in Sudan and determining which virus lineages may be present in this region will provide a more detailed understanding of the movement of virus strains and identification of areas at risk for CCHFV. We therefore analyzed an outbreak of hemorrhagic fever, including a nosocomial chain of transmission in a rural hospital in Sudan in 2008.

## The Study

In October 2008, an outbreak of hemorrhagic fever was reported in Al-fulah, Kordufan, Sudan. The index patient was a 60-year-old man who had worked as a butcher. The source of his infection was suspected to have been tissues and blood of an infected animal, although follow-up investigation was unable to precisely determine the source. He was admitted to a rural hospital with an acute febrile hemorrhagic illness after 3 days of high fever, chills, and headache. He had taken antimalarial medication at home, but his condition did not improve. He had epistaxis, black bloody vomitus, and diarrhea on the last 2 days of his illness. He died on day 5 after onset of illness.

No protective gloves or antiseptic products were available at the hospital. Illness developed in a male nurse who had provided care to the index patient 6 days after the index patient had been admitted to the hospital and in the chief male nurse a few days after that. The index patient’s sister was also considered to have a suspected case; she had sought care at the hospital after a heavy menstrual period that progressed to massive vaginal bleeding. The midwife who performed the gynecologic examination later became ill with high fever, vomiting of blood, and bloody diarrhea. As is tradition and social obligation in rural hospitals in this region, 2 relatives of the index patient had alternated caring for him (e.g., dressing him, changing his mattresses and bed sheets, nursing, and sleeping beside him) while he was in the hospital, and both acquired the infection (rapid onset of fever, headache, nausea, vomiting of blood, and bloody diarrhea). No details were available for 3 other patients with hemorrhagic fever associated with the hospital.

Of these 10 patients, 9 were admitted to a rural hospital in Al-fulah, where 6 continued to bleed, subsequently became comatose, and died. Records were unavailable for the other 3. In addition, 3 probable cases in the community were reported. Each of these 3 persons had a course of hemorrhagic disease and death that was compatible with CCHF; they had not been admitted to the hospital and could not be traced because of poor security conditions in the region. Patient ages varied from 15 to 70 years. Nosocomial transmission of the virus was likely the result of lack of personal protection for the hospital staff and the attending relatives, as has been often noted during previous outbreaks (*6*).

Of the patients for whom serum samples were available, 8 had evidence of acute CCHFV infection. Direct immunofluorescence assay detected no antibodies to CCHFV in any of the serum samples; however, all samples had been collected on days 1–3 of illness. Virus RNA extracted from each of the samples by QIAamp (QIAGEN GmbH, Hilden, Germany) was positive according to reverse transcription–PCR (RT-PCR) specific for CCHFV (*7*). The RNA was then used in RT-PCRs to amplify the entire virus S segment for complete sequencing as described (*8*). The full-length S segment nucleotide sequence of the strains from Sudan was 1,673 nt long, and the 8 viral sequences were identical with the exception of that from patient 4 (GenBank accession nos. GQ862371–2). A maximum-likelihood phylogenetic analysis of the S segment sequences placed the viruses from Sudan in group III (*8*), which is composed exclusively of viruses of African origin, including South Africa, Mauritania, and Nigeria (Figure). The highest nucleotide sequence identity was seen with strains from South Africa.

**Figure:**
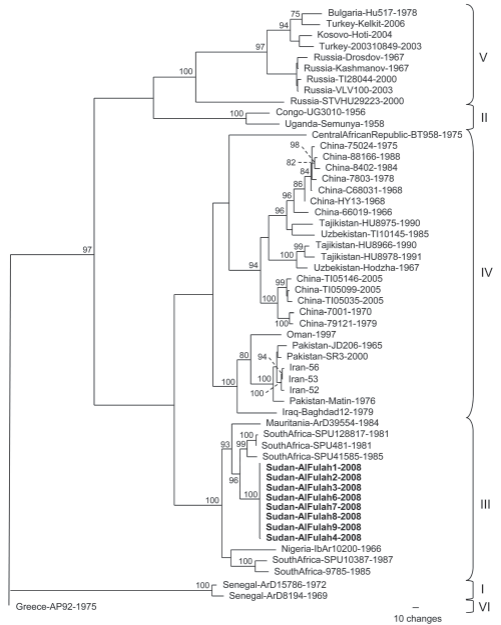
Phylogenetic relationship of Crimean-Congo hemorrhagic fever virus (CCHFV) full-length small (S) segments. Phylogenetic analysis used 47 full-length CCHFV S segments available in GenBank. GARLI (v0.96b8) (*9*) with default settings was used to generate a maximum-likelihood tree with bootstrap support values from 1,000 replicates. From the analysis, a 50% majority-rule tree was constructed. Virus strains from Sudan patients 1, 2, 3, 6, 7, 8, and 9 (GenBank accession no. GQ862371) were identical, and the virus sequence from patient 4 (GenBank accession no. GQ862372) differed by 1 nt. Each strain is listed by its location, strain name, and year of isolation, when available. **Boldface** indicates strains from Sudan; braces indicate previously described genetic lineages (*8*).

## Conclusions

Laboratory confirmation of 8 cases of CCHF demonstrates the presence of this disease in Sudan. Genetic analysis of the viruses showed that the strain involved was similar to strains found in South Africa, Mauritania, and Nigeria.

Detailed analysis of virus outbreaks is often limited by the lack of appropriate high-containment facilities required for virus isolation. However, appropriately sampled and stored acute-phase serum samples can have high titers of the virus, which enable extraction of virus RNA and genome sequencing studies without prior amplification of the virus in cell culture. In this study, serum specimens from 8 patients who died were positive for CCHFV by RT-PCR. Lack of virus-specific antibodies 1–3 days after onset of illness can be explained by the severity and rapid course of the disease, which does not allow sufficient time for antibody production. Virus-specific antibodies are, however, generally seen later in the course of illness and in persons who survive the infection.

Antibody studies have suggested the presence of various arboviruses in Sudan (*10**,**11*). Indirect evidence for CCHFV in animals in Sudan came from finding CCHFV-specific antibodies in animals imported from Sudan: camels in Egypt (*12*) and sheep and goats in Saudi Arabia (*13*). The finding that the Al-fulah outbreak was caused by a CCHFV strain from genetic group III illustrates how different virus strains and lineages can move with livestock transport or possibly bird migrations. The genome plasticity of the virus is surprisingly high for an arthropod-borne virus. This genetic diversity appears to be the result of not only accumulation of mutations but also of frequent RNA segment reassortment and even RNA recombination (*8* *14*,*15*,).

Clearly, this CCHFV is present in Sudan, and physicians should consider CCHF as a diagnosis for hemorrhagic fever cases in the region. In addition, efforts to provide appropriate personal protective supplies and training to medical staff in rural areas should be increased to help minimize risk for caregivers.
